# Factors influencing spinal anesthesia-to-delivery interval in elective cesarean sections: A retrospective analysis

**DOI:** 10.1097/MD.0000000000042420

**Published:** 2025-05-09

**Authors:** Michiko Kinoshita, Yoko Sakai, Yoshimi Nakaji, Rikako Takahashi, Yako Matsumoto, Katsuya Tanaka

**Affiliations:** a Department of Anesthesiology, Tokushima University Hospital, Tokushima-shi, Tokushima, Japan; b Division of Anesthesiology, Tokushima University Hospital, Tokushima-shi, Tokushima, Japan.

**Keywords:** anesthesia, cesarean section, fetal blood, fetal distress, spinal

## Abstract

Previous research has shown that longer intervals from spinal anesthesia (SA) to cesarean delivery are associated with lower umbilical arterial pH levels. This study aimed to identify specific risk factors related to SA-to-delivery interval in elective cesarean sections. In this retrospective analysis, 404 singleton parturients who underwent elective cesarean sections with SA after 37 weeks of gestation were assessed. Factors influencing the SA-to-delivery interval were explored. The correlation between SA-to-delivery interval and umbilical arterial pH levels was also investigated in our cohort. Factors that significantly prolonged the SA-to-delivery interval included higher body mass index (BMI; regression coefficient [RC]: 0.206, *P* = .003), history of previous cesarean section (RC: 1.699, *P* = .012), placenta previa/low-lying placenta (RC: 6.141, *P* < .001), and local anesthetic administration into the epidural space following SA (RC: 3.279, *P* < .001). The SA-to-delivery interval was slightly yet significantly inversely correlated with umbilical arterial pH (*r* = −0.163, *P* = .001). A general linear model, adjusted for age, BMI, predominant ephedrine use, lowest systolic blood pressure between SA and delivery, diabetes mellitus, and hypertensive disorder of pregnancy have established the significant relationship between the SA-to-delivery interval and umbilical arterial pH (RC: −0.0012, *P* < .001). In conclusion, factors such as higher BMI, previous cesarean sections, placenta previa/low-lying placenta, and local anesthetic administration into the epidural space after SA significantly contributed to extended SA-to-delivery intervals. The SA-to-delivery interval was inversely correlated with umbilical arterial pH.

## 1. Introduction

Cesarean delivery rates have increased globally, with Japan reporting approximately 20%, the United States and China each exceeding 30%, and the worldwide rate surpassing 20%.^[1-4]^ Spinal anesthesia (SA) is primarily employed for cesarean deliveries because it provides a rapid onset of effective anesthesia while minimizing fetal exposure to anesthetic agents and maternal risks associated with general anesthesia.^[5,6]^ Despite its safety profile, neonates born via cesarean deliveries under SA may be at risk of hypoxia.^[7,8]^ Understanding the factors contributing to adverse fetal outcomes is crucial to enhance neonatal safety in cesarean deliveries with SA.

The intervention-to-delivery interval has traditionally been emphasized in emergency cases but not in elective procedures. Recent evidence, however, has challenged this view.^[9]^ Hypotension frequently occurs following SA, with both its cumulative duration and severity being associated with lowered Apgar scores and fetal acidemia.^[10,11]^ A study in 2019 first revealed that despite a stable fetus in category-4 elective cesarean sections, longer intervals from SA to delivery were associated with decreased umbilical arterial pH, which was also supported by subsequent research.^[12-14]^ This highlights the necessity of addressing prolonged intervals from SA to delivery not only in emergency scenarios but also in nonemergency cases. While factors affecting intervention-to-delivery time have been studied extensively in emergency cesarean sections,^[15-18]^ risk factors influencing this interval in elective procedures under SA remain insufficiently explored.

To address this gap, we aimed to identify specific risk factors for prolonged SA-to-delivery intervals in elective cesarean sections. Additionally, we investigated the correlation between these prolonged intervals and umbilical arterial pH levels in our patient cohort.

## 2. Materials and methods

### 2.1. Study design and ethics

This retrospective observational study was reviewed and received approval from the Ethics Committee of Tokushima University Hospital (approval number: 4447) and registered with the University Hospital Medical Information Network Clinical Trial Registry (UMIN000052821, registration date: November 25, 2023). Although the patient cohort partially overlapped with our previous research,^[19,20]^ this study was conducted independently, and its separate publication was approved by the Ethics Committee. The requirement for written informed consent for all patients was waived, which was publicized on our department’s website using an opt-out approach. This manuscript adheres to the Strengthening the Reporting of Observational Studies in Epidemiology Statement.

### 2.2. Participants

Singleton parturients who underwent selective cesarean sections with SA at Tokushima University Hospital between May 2019 and October 2023 were included. The study period was determined according to the implementation date of the current electronic anesthesia record system at the hospital. Exclusion criteria were as follows: emergency cases, cesarean delivery under general anesthesia, gestational age <37 weeks, multiple gestation, and intrauterine fetal death.

### 2.3. Facility-standard anesthesia technique

At our institution, elective cesarean sections are generally performed by combining spinal and epidural anesthesia. Initially, an epidural catheter is placed in the lower thoracic region. Subsequently, SA is administered with 0.5% hyperbaric bupivacaine. According to the anesthesiologist’s discretion, either fentanyl (10–20 μg) or morphine (100–200 μg) may be incorporated. As customary in our facility, the instance of intrathecal injection is documented as the time of SA in the anesthesia record. Primarily, SA targets surgical anesthesia, while epidural anesthesia is reserved for postoperative pain management. However, for inadequate SA, levobupivacaine, ropivacaine, or mepivacaine can be administered epidurally via the preplaced catheter. To optimize efficiency during SA, the surgeon undertakes preparatory steps, including surgical scrubbing.

### 2.4. Data collection and outcomes

Demographic, obstetric, newborn, and intraoperative data were retrospectively collected from medical records, obstetric delivery database, and anesthesia records. As a preliminary investigation, we examined whether the longer interval from SA to delivery was associated with lower umbilical arterial pH in our sample data, as in previous studies. Furthermore, umbilical arterial base excess and Apgar score were also assessed. This study focused on examining factors contributing to the interval from SA to delivery.

### 2.5. Statistical analysis

Data are presented as means (standard deviation) or numbers (percentages). A general linear mixed model was utilized to assess potential factors influencing the SA-to-delivery interval, with individual surgeons considered as a random effect. In this analysis, variables exhibiting *P* < .1 in univariate analysis (using Welchs *t*-test, analysis of variance, and Pearsons correlation coefficient) were included. Regardless of univariate analysis outcomes, the following candidate independent variables, as referred from previous literature, were also incorporated: body mass index (BMI), history of prior cesarean section, and fetal birthweight.^[15,21,22]^ For general linear (mixed) models, the number of explanatory variables was fewer than one-tenth of cases. Pearson’s correlation coefficient was utilized for examining the relationship between SA-to-delivery interval and umbilical arterial blood gas analysis. The independent effect of the SA-to-delivery interval on umbilical arterial blood gas analysis was assessed via a general linear model adjusting for potential confounding factors: maternal age, BMI, predominant ephedrine use, lowest systolic blood pressure before delivery, diabetes mellitus, and hypertensive disorder of pregnancy. These factors were selected before the analysis, according to previous reports.^[11,23-26]^ In accordance with a previous report, predominant ephedrine use was defined as a higher proportion of ephedrine compared to phenylephrine in vasopressors used intraoperatively, assuming 8 mg ephedrine to be approximately equipotent to 100 μg phenylephrine.^[27]^

Statistical analyses were conducted using R version 4.2.3 (The R Foundation for Statistical Computing, Vienna, Austria) and EZR (Saitama Medical Center, Jichi Medical University, Saitama, Japan).^[28]^ A 2-sided *P* < .05 was considered statistically significant.

## 3. Results

**Figure 1.**
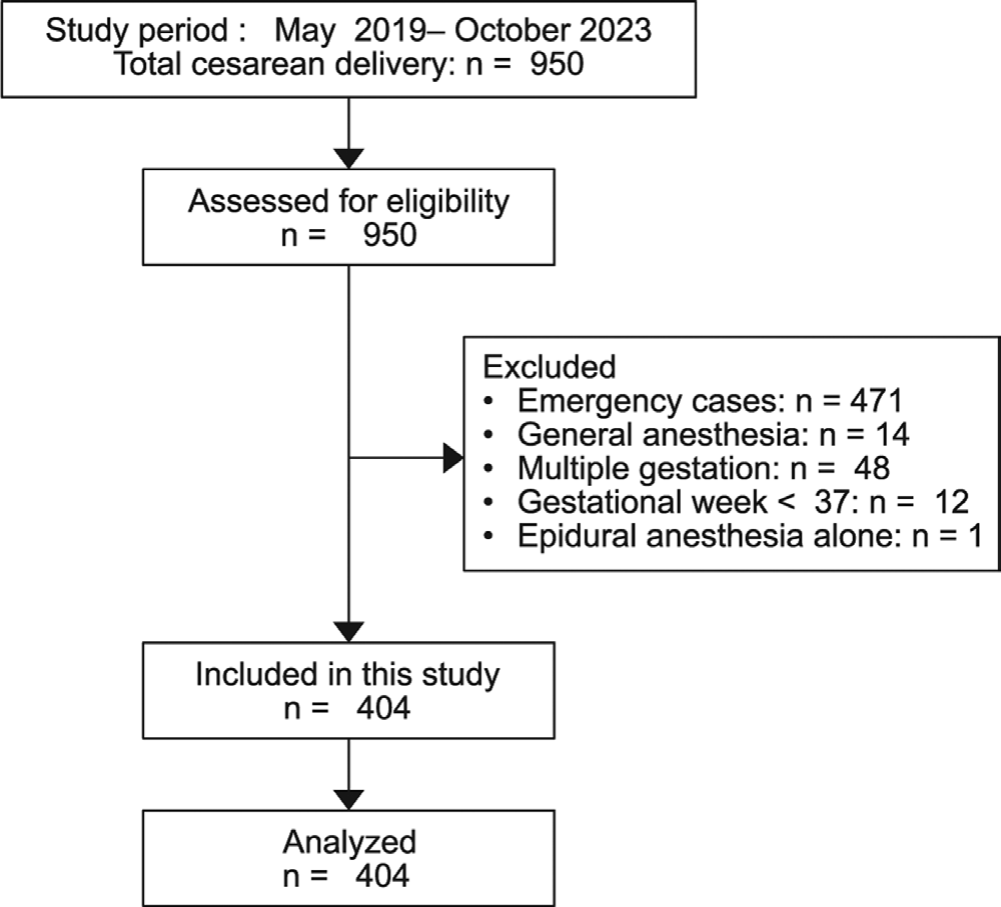
Flow diagram.

A total of 950 cesarean deliveries were identified during the study period. Using the eligibility and exclusion criteria, 404 cases were analyzed. A flow diagram is presented in Figure 1. Demographic, obstetric, newborn, and intraoperative data are presented in Table 1.

**Table 1:** Demographic, obstetric, newborn, and intraoperative data (n = 404).

Demographic data	
Age (yr)	34.5 (4.7)
BMI (kg/m^2^)	25.1 (4.4)
Obstetric data	
Gestational (wk), 37/38/39	70 (17.3)/332 (82.2)/2 (0.5)
Assisted reproductive technology (n, %)	76 (18.8)
Previous cesarean section (n, %)	273 (67.6)
Placenta previa/low-lying placenta (n, %)	55 (13.6)
Fetal position, cephalic/breech/transverse (n, %)	324 (80.2)/77 (19.1)/3 (0.7)
Diabetes mellitus (n, %)	48 (11.9)
HDP (n, %)	23 (5.7)
Newborn data	
Fetal birthweight (g)	2913 (380)
1-min Apgar score	7.9 (0.9)
5-min Apgar score	9.2 (0.6)
Umbilical arterial pH	7.28 (0.04)
Umbilical arterial base excess (mmol/L)	−2.15 (2.19)
Intraoperative data	
Dose of 0.5% bupivacaine (mg)	9.59 (0.73)
Opioid addition to bupivacaine (n, %)	369 (91.3)
Local anesthetic administration into the epidural space after SA (n, %)	84 (20.8)
Lowest SBP between SA and delivery (mm Hg)	89.0 (14.9)
Predominant ephedrine use (n, %)	57 (14.1)
SA-to-delivery interval (min)	22.6 (6.3)
SA-to-skin incision interval (min)	13.0 (4.8)
Skin incision-to-delivery interval (min)	9.7 (5.0)

Data are expressed as mean (standard deviation) or number (percentage).

BMI = body mass index, HDP = hypertensive disorder of pregnancy, SA = spinal anesthesia, SBP = systolic blood pressure.

Table 2 illustrates the univariate analysis for the SA-to-delivery interval. Factors such as placenta previa/low-lying placenta, opioid administration to SA, and local anesthetic administration into the epidural space following SA were significantly associated with a prolonged interval from SA to delivery.

**Table 2:** Univariate analysis for the SA-to-delivery interval.

Variables	*P*-value
Demographic data	
Age (yr)	.903
BMI (kg/m^2^)	.071
Obstetric data	
Gestational (wk), 37/38/39	.142
Assisted reproductive technology (binary)	.440
Previous cesarean section (binary)	.623
Placenta previa/low-lying placenta (binary)	<.001
Fetal position, cephalic/breech/transverse	.438
Diabetes mellitus (binary)	.939
HDP (binary)	.159
Newborn data	
Fetal birthweight (g)	.210
Intraoperative data	
Dose of 0.5% bupivacaine (mg)	.721
Opioid addition to SA (binary)	.004
Local anesthetic administration into the epidural space following SA (binary)	<.001

BMI = body mass index, HDP = hypertensive disorder of pregnancy, SA = spinal anesthesia.

Table 3 summarizes the general linear mixed model, incorporating individual surgeons as a random effect, with the interval from the SA to delivery as the dependent variable. Factors significantly prolonging the SA-to-delivery interval included: BMI (regression coefficient [RC]: 0.206, 95% confidence interval [CI]: 0.073–0.338, *P* = .003); history of previous cesarean section (RC: 1.699, 95% CI: 0.396–3.020, *P* = .012); placenta previa/low-lying placenta (RC: 6.141, 95% CI: 4.236–7.996, *P* < .001); and local anesthetic administered into the epidural space after SA (RC: 3.279, 95% CI: 1.889–4.675, *P* < .001). Of which, the history of previous cesarean section (RC: −1.003, 95% CI: −1.899 to −0.119, *P* = .029), placenta previa/low-lying placenta (RC: 6.481, 95% CI: 5.245–7.718, *P* < .001), and local anesthetic administered into the epidural space after SA (RC: 2.959, 95% CI: 2.013–3.906, *P* < .001) were significantly associated with the interval from SA to skin incision, as summarized in Table S1, Supplemental Digital Content, https://links.lww.com/MD/O897. Furthermore, BMI (RC: 0.120, 95% CI: 0.015–0.226, *P* = .027) and history of previous cesarean section (RC: 2.805, 95% CI: 1.765–3.859, *P* < .001) were significantly associated with the interval from the skin incision to delivery, as summarized in Table S2, Supplemental Digital Content, https://links.lww.com/MD/O898.

**Table 3:** General linear mixed model for the SA-to-delivery interval.

Variables	Regression coefficient	95% CI	*P*-value
BMI (kg/m^2^)	0.206	0.073 to 0.338	.003
Previous cesarean section (binary)	1.699	0.396 to 3.020	.012
Placenta previa/low-lying placenta (binary)	6.141	4.236 to 7.996	<.001
Fetal birthweight (g)	−0.001	−0.003 to 0.000	.070
Opioid addition to SA (binary)	1.899	−0.088 to 3.904	.064
Local anesthetic administration into the epidural space following SA (binary)	3.279	1.889 to 4.675	<.001

BMI = body mass index, CI = confidence interval, SA = spinal anesthesia.

**Figure 2.**
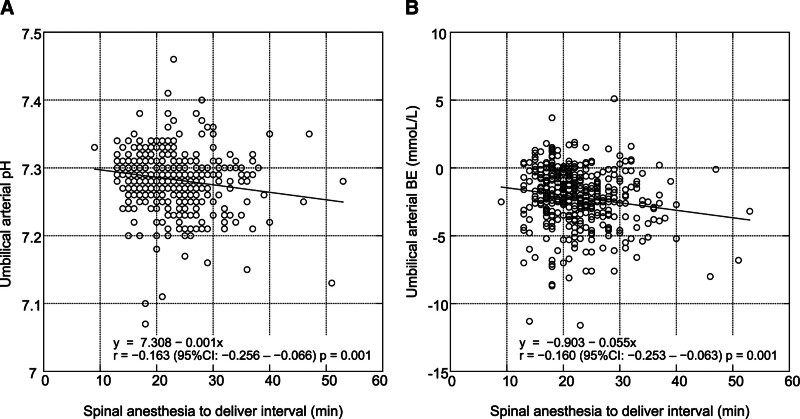
Correlation between spinal anesthesia-to-delivery interval and umbilical arterial pH (A)/base excess (B). BE = base excess, CI = confidence interval.

The interval from SA to delivery was slightly yet significantly inversely correlated with both umbilical arterial pH and base excess (*r* = −0.163, *P* = .001; *r* = −0.160, *P* = .001; respectively), as shown in Figure 2. A general linear model, incorporating adjustments for age, BMI, predominant ephedrine use, lowest systolic blood pressure between SA and delivery, diabetes mellitus, and hypertensive disorder of pregnancy, confirmed the significant relationship between the interval from SA to delivery and both umbilical arterial pH and base excess (RC: −0.0012, 95% CI: −0.0018 to −0.0005, *P* < .001; −0.056, 95% CI: −0.089 to −0.022, *P* = .001; respectively; Table 4). No significant association was identified between the interval from SA to delivery and both the 1- and 5-min Apgar scores (*R* = 0.008, *P* = .865; *R* = 0.049, *P* = .325; respectively).

**Table 4:** General linear model for umbilical arterial pH and base excess.

Variables	Regression coefficient	95% CI	*P*-value
Umbilical arterial pH			
Age (yr)	0.0006	−0.0003 to 0.0015	.213
BMI (kg/m^2^)	−0.0002	−0.0012 to 0.0008	.702
Ephedrine dominant use (binary)	0.0002	−0.0118 to 0.0121	.978
Lowest SBP between SA and delivery (mm Hg)	0.0003	0.0000 to 0.0006	.032
Diabetes mellitus (binary)	0.0011	−0.0122 to 0.0146	.863
HDP (binary)	−0.0108	−0.0289 to 0.01456	.245
SA-to-delivery interval (min)	−0.0012	−0.0018 to −0.0005	<.001
Umbilical arterial base excess (mmol/L)			
Age (yr)	0.022	−0.024 to 0.065	.368
BMI (kg/m^2^)	−0.031	−0.080 to 0.020	.229
Ephedrine dominant use (binary)	0.322	−0.279 to 0.924	.293
Lowest SBP between SA and delivery (mm Hg)	0.007	0.002 to 0.032	.022
Diabetes mellitus (binary)	−0.530	−1.205 to 0.145	.124
HDP (binary)	−0.361	−1.277 to 0.555	.439
SA-to-delivery interval (min)	−0.056	−0.089 to −0.022	.001

BMI = body mass index, CI = confidence interval, HDP = hypertensive disorder of pregnancy, SA = spinal anesthesia, SBP = systolic blood pressure.

## 4. Discussion

This study identified specific risk factors associated with the interval from SA to delivery in elective cesarean sections. Higher BMI, previous cesarean section, placenta previa/low-lying placenta, and local anesthetic administration into the epidural space following SA were found to significantly extend this interval. Furthermore, we confirmed a negative correlation between the SA-to-delivery interval and umbilical arterial pH levels.

Among the risk factors we identified, higher BMI and previous cesarean section are consistent with findings from previous studies.^[15,16,18,21,29,30]^ A higher BMI may lead to a longer physical distance to access the fetus from the body surface, while a previous cesarean section may induce intraperitoneal adhesions, potentially complicating the surgical procedure.^[29-32]^ Our findings confirm that these risk factors apply to elective cesarean sections, which can help improve planning for these procedures.

Our analysis newly identified placenta previa and low-lying placenta as significant factors that delayed the initial skin incision following SA, contributing to prolonged time to delivery. This delay appears to be associated with preoperative preparations for uterine balloon tamponade (Bakri balloon) insertion, a procedure aimed at reducing postpartum hemorrhage risk.^[33,34]^ While this technique sometimes requires additional preventive measures such as cervical stabilization to prevent balloon displacement,^[35,36]^ we recommend that these preparatory steps are conducted as quickly as possible to minimize the SA-to-delivery interval.

Our study further demonstrated that the administration of additional local anesthetic into the epidural space following SA was associated with a significant prolongation of the time to delivery. This supplemental anesthesia was presumably necessary owing to insufficient efficacy of the initial spinal block. Previous studies have identified the ED50 and ED95 for intrathecal hyperbaric bupivacaine with opioids as 6.0 and 12.6 mg, respectively. The mean bupivacaine dose in our cohort was 9.59 mg, which may have been inadequate.^[37]^ Rimsza et al demonstrated that a prolonged interval from the initiation of SA, rather than from the time when adequate anesthesia was achieved, was correlated with a decrease in umbilical arterial pH.^[12]^ Considering Rimsza et als findings,^[12]^ we recommend initially administering a sufficient dose of anesthetic to ensure effective SA to prevent delivery delays.

Our analysis demonstrated a negative correlation between the SA-to-delivery interval and umbilical artery pH, consistent with findings from previous research.^[12-14]^ While the exact mechanism remains uncertain, the cumulative duration of SA-induced placental hypoperfusion may have compromised fetal oxygenation. Knigin et al have demonstrated that anesthesia-induced hypotension and anesthesia-to-delivery interval have independent and additive impacts on umbilical arterial pH reduction.^[13]^ This indicates that meticulous control of maternal blood pressure alone is insufficient to mitigate fetal distress; efforts to minimize the anesthesia-to-delivery interval are also required.

This study has some limitations. First, the retrospective nature of data collection may potentially lead to inaccuracies, rendering it less reliable than data from prospectively designed studies. The timings of SA and delivery are dependent on the accuracy of medical/anesthetic records. Furthermore, there is a possibility of incomplete records of the parturients’ preexisting medical conditions. Second, since this study was conducted at a single university hospital in Japan, its results may have limited generalizability owing to potential biases towards specific regions and demographics. Third, although it is conceivable that fetal delivery times vary between inexperienced and experienced surgeons, this aspect was not analyzed further. Particularly in an academic setting, the rationale for this omission is the potential for senior surgeons to allocate additional time for instructional purposes during cesarean sections. Furthermore, bias may arise since senior surgeons are often assigned to more challenging surgeries. Consequently, surgeon-specific variations were treated as random effects within a general linear mixed model. Fourth, many previous studies have utilized pH values below 7.1 or 7.2 as a clinically significant threshold.^[12-14,21,25,38,39]^ However, our dataset included very few instances of values falling below these pH levels (1 case below pH 7.1, and 7 cases between pH 7.1 and 7.2), precluding a meaningful analysis related to this criterion.

In conclusion, factors such as high BMI, previous cesarean sections, placenta previa/low-lying placenta, and local anesthetic administration to the epidural space were identified as contributors to an extended interval from SA to delivery. Given that prolonged SA-to-delivery intervals can cause fetal distress, it is crucial for clinicians to be aware of these risk factors and actively strive for rapid fetal delivery, even in elective cesarean sections.

## Acknowledgments

As non-native English speakers, we exclusively used ChatGPT (GPT-4, OpenAI, San Francisco) to improve the clarity and accuracy of the English text. We thoroughly reviewed and verified the accuracy of the suggestions provided by the artificial intelligence before incorporation. All statements related to this study’s hypotheses, interpretations, results, conclusions, limitations, and implications represent original ideas and work. The final version of the manuscript has been professionally edited by a native English speaker and an editor from a Manuscript editing company (Editage).

## Author contributions

**Conceptualization:** Michiko Kinoshita, Yoko Sakai, Katsuya Tanaka.

**Formal analysis:** Michiko Kinoshita.

**Investigation:** Michiko Kinoshita, Yoko Sakai, Yoshimi Nakaji, Rikako Takahashi, Yako Matsumoto.

**Methodology:** Michiko Kinoshita, Yoko Sakai, Yoshimi Nakaji, Rikako Takahashi, Yako Matsumoto.

**Project administration:** Katsuya Tanaka.

**Supervision:** Katsuya Tanaka.

**Writing – original draft:** Michiko Kinoshita.

**Writing – review & editing:** Yoko Sakai, Yoshimi Nakaji, Rikako Takahashi, Yako Matsumoto, Katsuya Tanaka.

## Supplementary Material


