# Supplementary material for: Factors influencing spinal anesthesia-to-delivery interval in elective cesarean sections: A retrospective analysis
Source: Medicine (Baltimore). 2025 May 9;104(19):e42420. doi: 10.1097/MD.0000000000042420 (PMC12074038; doi:10.1097/MD.0000000000042420)
Supplement: Supplementary file 1 [file medi-104-e42420-s001.pdf]

Supplemental Table S1: General linear mixed model for the SA-to-skin incision interval

Variables	Regression coefficient	95% CI	*p*-value
BMI (kg/m^2)	0.082	−0.008 to 0.172	0.076
Previous cesarean section (binary)	−1.003	−1.899 to −0.119	0.029
Placenta previa/low-lying placenta (binary)	6.481	5.254 to 7.718	<0.001
Fetal birthweight (g)	−0.001	−0.002 to 0.001	0.322
Opioid addition to SA (binary)	0.838	−0.514 to 2.199	0.229
Local anesthetic administration into the epidural space following SA (binary)	2.959	2.013 to 3.906	<0.001

BMI, body mass index; CI, confidence interval; SA, spinal anesthesia.
