# Supplementary material for: Factors influencing spinal anesthesia-to-delivery interval in elective cesarean sections: A retrospective analysis
Source: Medicine (Baltimore). 2025 May 9;104(19):e42420. doi: 10.1097/MD.0000000000042420 (PMC12074038; doi:10.1097/MD.0000000000042420)
Supplement: Supplementary file 2 [file medi-104-e42420-s002.pdf]

Supplemental Table S2: General linear mixed model for the skin incision-to-delivery interval

Variables	Regression coefficient	95% CI	*p*-value
BMI (kg/m^2)	0.120	0.015 to 0.226	0.027
Previous cesarean section (binary)	2.805	1.765 to 3.859	<0.001
Placenta previa/low-lying placenta (binary)	−0.299	−1.816 to 1.184	0.696
Fetal birthweight (g)	−0.001	−0.002 to 0.000	0.121
Opioid addition to SA (binary)	1.197	−0.390 to 2.795	0.143
Local anesthetic administration into the epidural space following SA (binary)	0.450	−0.661 to 1.562	0.431

BMI, body mass index; CI, confidence interval; SA, spinal anesthesia.
